# Multi-layer network utilizing rewarded spike time dependent plasticity to learn a foraging task

**DOI:** 10.1371/journal.pcbi.1005705

**Published:** 2017-09-29

**Authors:** Pavel Sanda, Steven Skorheim, Maxim Bazhenov

**Affiliations:** 1 Department of Medicine, University of California, San Diego, La Jolla, California, United States of America; 2 Information and Systems Sciences Lab, HRL Laboratories, LLC, Malibu, California, United States of America; Research Center Jülich, GERMANY

## Abstract

Neural networks with a single plastic layer employing reward modulated spike time dependent plasticity (STDP) are capable of learning simple foraging tasks. Here we demonstrate advanced pattern discrimination and continuous learning in a network of spiking neurons with multiple plastic layers. The network utilized both reward modulated and non-reward modulated STDP and implemented multiple mechanisms for homeostatic regulation of synaptic efficacy, including heterosynaptic plasticity, gain control, output balancing, activity normalization of rewarded STDP and hard limits on synaptic strength. We found that addition of a hidden layer of neurons employing non-rewarded STDP created neurons that responded to the specific combinations of inputs and thus performed basic classification of the input patterns. When combined with a following layer of neurons implementing rewarded STDP, the network was able to learn, despite the absence of labeled training data, discrimination between rewarding patterns and the patterns designated as punishing. Synaptic noise allowed for trial-and-error learning that helped to identify the goal-oriented strategies which were effective in task solving. The study predicts a critical set of properties of the spiking neuronal network with STDP that was sufficient to solve a complex foraging task involving pattern classification and decision making.

## Author summary

This study explores how intelligent behavior emerges from the basic principles known at the cellular level of biological neuronal network dynamics. Compared to the approaches used in the artificial intelligence community, we applied biologically realistic modeling of neuronal dynamics and plasticity. The building blocks of the model are spiking neurons, spike-time dependent plasticity (STDP) and homeostatic rules, known experimentally, which are shown to play a fundamental role in both keeping the network stable and capable of continous learning. Our study predicts that a combination of these principles makes possible a foraging behavior in a previously unknown environment, including pattern classification to distinct between environment shapes which are rewarded and those which are punished and decision making to select the optimal strategy to acquire the maximal number of the rewarded elements. To solve this complex task we used multi-layer neuronal processing that implemented pattern generalization by unsupervised STDP at the earlier processing step, as commonly observed in the animal and human sensory processing, followed by reinforcement learning at the later steps. In the model, the intelligent behavior emerged spontaneously due to the network organization implementing both local unsupervised plasticity and reward feedback resulting from a successful behavior in the environment.

## Introduction

Biologically inspired neural networks should be capable of performing sophisticated information processing that the human and animal brains can perform. Information processing by the brain is deeply multilayered and involves many sequential steps before sensory information can be interpreted and translated into behavior. What makes this cascade powerful and unique is its capability to learn and respond to an ever changing environment. In the most studied sensory pathway—the visual one—the sensory input gets progressively more general, though the stages at which visual learning occurs are still a matter of controversy and different plasticity mechanisms might be operating at different processing steps [1]. Eventually sensory information reaches decision centers (such as lateral intraparietal cortex) which govern behavior and those centers are under the influence of reward signals [2, 3]. It has been shown that reinforcement based on the reward can be vital to visual learning [4]. To what extent reward influences learning in the sensory cortices seems to be task dependent and different studies showed both learning with [5, 6] and without [7, 8] the presence of reward.

While a great deal of research has gone into understanding the mechanisms of learning, it is still not fully known how learning at the cellular level gives rise to the learning at the level of animal behavior. Most learning models successfully employ hebbian type learning principles formulated as an abstract rule [9] without being concerned with the cellular level mechanisms. The most promising model of learning at the synaptic level is spike time dependent plasticity (STDP) [10]. In STDP, when a presynaptic cell fires shortly before a post synaptic cell, the synapse between them increases in strength. If the postsynaptic cell fires before the presynaptic cell, the synapse decreases in strength. Such basic form of STDP is only capable of unsupervised learning. By storing STDP events in the form of synaptic tags [11], a delayed reward signal can enable a network to perform reinforcement learning [12]. This mechanism relies on the dopamine modulation of synaptic tags created by STDP [13, 14, 15]. A small number of studies have attempted to perform task learning using rewarded STDP including studies that attempted to maintain the stability of a virtual robotic arm [16, 17].

In theoretical studies, STDP commonly leads to the net increase of synaptic strength across the network [18], causing large numbers of synapses to climb toward their maximum values. This would result in a highly dysfunctional brain and can eventually promote an epileptic state [19]. To prevent runaway synaptic dynamics, various regulatory synaptic mechanisms maintain the distribution of synaptic weights in biological networks. This includes homeostatic mechanisms when a group of cells that experiences high firing frequencies over a prolonged period becomes less responsive to excitatory input by effectively reducing incoming excitatory synaptic weights [20]. Another regulatory mechanism, heterosynaptic plasticity, responds to the increase of a synaptic weight by reducing all other incoming synaptic strengths of the same post synaptic neuron [21, 22, 23, 24] preventing neurons from becoming overactive [25, 26, 27, 28].

In this new work, we built a model containing two plastic layers inspired by the pathways known in mammalian brain from sensory to higher order sensory-motor areas responsible for decision making [29, 4]. The first layer of the model uses unsupervised (unrewarded) learning to classify the input while the second layer (based on rewarded STDP) is responsible for decision making. As a whole the network simulated the brain of an agent moving through an unknown environment, continuously learning distinct input patterns of food and adjusting synaptic weights controlling its movement according to the reward and punishment signals based on the shape of the different configurations of food particles that it acquires.

We demonstrate that such multilayered network combining rewarded and nonrewarded STDP and synaptic regulatory mechanisms is capable of solving a more complex foraging task, involving discrimination between elementary patterns of food, than it was previously reported in the networks with only one plastic layer based on rewarded STDP [30].

## Results

### The organization of the foraging neural network

**Fig 1:**
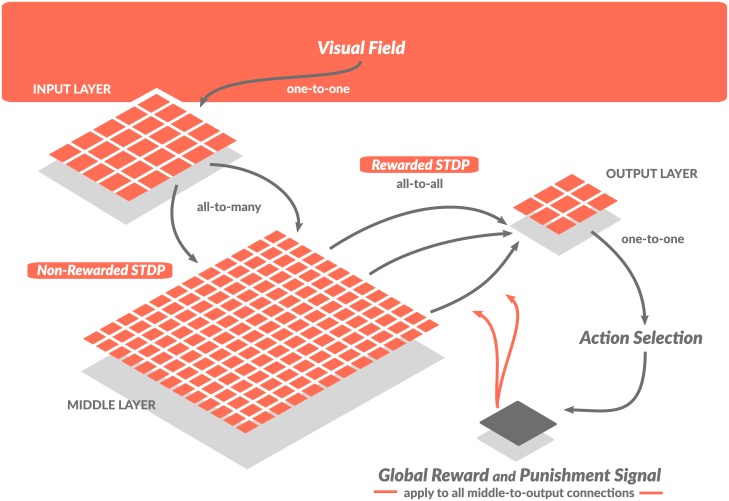
Network organization. The network organization is a simplification of the information processing flow known in the visual pathway, involving mapping of the sensory input into the higher level representations and then using them for decision making in the prefrontal cortex [4]. Input indicating the position of food particles relative to the virtual agent (positioned in the center of the field) was simulated as a set of excitatory inputs to the input layer neurons. In the model, each input layer cell sends one excitatory and one inhibitory connection to each of the cells in the middle layer where object representation is built. Each middle layer cell sends one excitatory and one inhibitory connection to 9 cells in the output layer. The most active cell in the output layer (size 3x3) decides the direction of subsequent movement. Excitatory connections from the input to the middle layer are subject to non-rewarded STDP. Excitatory connections from the middle layer to the output layer are subject to rewarded STDP where reward depends on whenever a move results in food acquisition. Inhibitory connections from a given cell always match the average strength of the excitatory outputs of the same presynaptic cell.

In this study we have expanded upon a network with a single plastic layer which was designed to learn and perform a foraging task in a virtual environment [30]. The new model incorporated two layers of plastic synaptic connections and implemented both rewarded and non-rewarded spike time dependent plasticity (STDP), see Fig 1. It included one-to-many connections from the input layer to the large middle layer and all to all connections from the middle layer to the output layer. Each middle layer cell received inputs from a few randomly selected input neurons. Reducing the number of inputs to each middle layer cell greatly reduced the computational power required for the simulation and was also a realistic approximation of the brain anatomy [31]. Connections between all three layers were set up initially at random and as such any correlation between the input pattern and the network response was random. As the simulation progressed, the learning in the form of changes in synaptic weights caused the network to become more proficient in obtaining food. Connections from the input to the middle layer employed non-rewarded STDP. This form of unsupervised learning allowed the network to respond more readily to the common features (such as spatial patterns), and common combinations of features, within the environment. Connections from the middle to output layers were controlled by the rewarded STDP. Thus, STDP traces were recorded as synaptic tags [13] and only applied if a reward signal was received later. The strength of traces declined, with time causing older traces to have less impact if a reward was eventually applied. A global reward signal was received whenever the network directed the virtual agent to a goal (”food” location). Synapses that observed pre-before-post combination of the action potentials within a few movement cycles before the reward became stronger; therefore, it increased the probability for the same postsynaptic cell to spike when the same input occurred in the future. The supervised learning allowed the network to develop approach or avoidance behavior based on the reward predicting value of the stimuli.

To evaluate the training performance, we compared our STDP based learning algorithm to a set of simple heuristic algorithms. In the best performing heuristic algorithm, when no food was present in the visual field, the agent either continued in the same direction it moved on the last step with a 98% probability or turned at 45 degrees left or right with a 2% probability. When the food was present, the heuristic algorithm searched through all possible combinations of 5 moves within the visual field. It then chose the set of moves that would result in the most food being obtained and made the first move from that set. If multiple sets of moves obtained the same number of food particles, the set which obtained food sooner was selected. If multiple sets had the same sequence of food being obtained, one of those sets was chosen randomly. Under standard conditions (such as given density of food particles in the environment) this strategy had an average food acquisition rate of 56%. Our multilayered network combining rewarded and nonrewarded STDP was able to achieve very close acquisition rate of 52%. For comparison, in the previous study we were able to achieve average food acquisition rates of about 48% [30].

This paper is divided into two main sections. In the first part we considered a simple task to obtain food at any location regardless of the spatial pattern of the food particles. In the second part, we increased the complexity of the task, so the agent was trained to pick up food organized within a specific spatial pattern (horizontal bar) and avoid any food particles organized within another pattern (vertical bar). The last task could not be achieved by the simpler network with only one plastic layer [30]. Thus, the main achievement of the current model is that it sets the stage for further development into the learning of more complex tasks, however, it also shows a modest improvement compared to the simpler models performing with food acquisition rates above 51%.

### Simple foraging behavior in the model with two plastic layers

**Fig 2:**
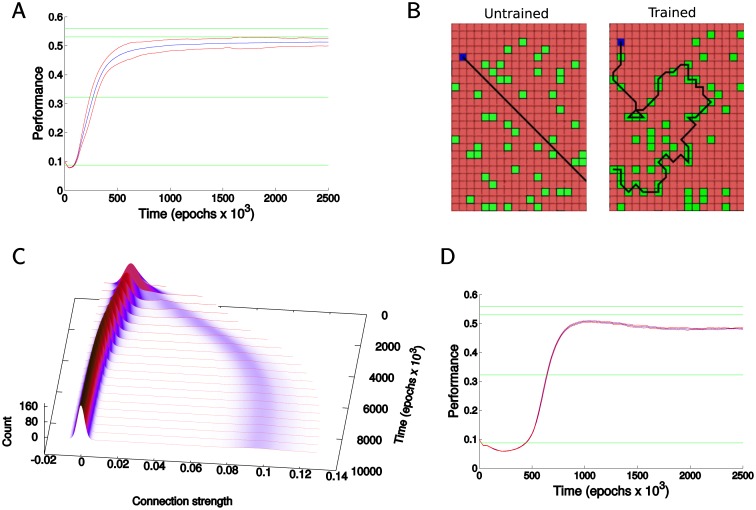
Basic two plastic layers model. A) Dynamics of the network performance over time. The Y axis is the rate of food acquisition per move as an exponential moving average. The X axis is time in epochs (each epoch consists of 600 time steps). Final food acquisition rates often exceeded 52% which is higher than in the previous models [30] and performance was very reliable across trials. Blue lines represent the mean performance and red lines represent the mean the standard deviation. Green lines represent performance of heuristic algorithms—lowest: random movement (98% go straight, 2% turn); second to lowest: as the lowest, but move to any food if directly adjacent; third to lowest: move toward closest food among all particles within the visual field; highest: try all possible combinations within the visual field, then take the first move of most successful set. B) Examples of the agent movement through the environment. Left before training, right after ∼120 × 10^3^ training steps. C) Time evolution of the strength of excitatory connections from the input to the middle layer during the first 10000 epochs (the profile at any point in time corresponds to the histogram of strengths). Note a group of synapses that significantly increased their strength. D) Performance of the model without normalization of synaptic potentiation based on spike rate. Note that the model showed declines in performance occurring after initial peak in all of the trials and over a wide range of testing parameters. Green lines as in A.

In Fig 2 we show the dynamics of a complete model for a simple foraging task of food acquisition regardless of the spatial arrangement of the food particles. Various homeostatic mechanisms were applied in order to obtain the best performance for a simple foraging task of acquiring food; the contribution of individual mechanisms will be discussed in detail in the following sections. Over time performance increased (Fig 2A) as the agent learned to move into the direction of food (Fig 2B). The Fig 2C shows the dynamics of the strengths of excitatory connections from the input to the middle layer over time. As the network learned a task, a small fraction of synapses increased and moved apart from the majority of other synapses which were slightly reduced. This small subset of synapses played the decisive role in learning, and maintaining their size and stable distance from the bulk of synapses kept the network behavior stable.

#### Normalization of synaptic potentiation

In the early versions of the model we observed a small but consistent decline in performance over time after performance reached its peak (see Fig 2D). This dynamic seems to be an inherent property of the networks that experience a positive net reward value. Indeed, synapses that are regularly active would have an advantage in gaining synaptic strength regardless of whether their activation improves the probability of reward or not. We implemented a new normalization mechanism to counteract this effect. The average value of STDP traces created by the synapse over an extended period (thousands of cycles) was recorded for each synapse between the middle and the output layer. The magnitude of each STDP trace at a synapse was then normalized by this average value before being applied as a change in synaptic strength. Thus a synapse that regularly experienced strong traces from STDP events, had progressively reduced potentiation from new STDP events. As a result, the slow decline in network performance was completely eliminated and significant improvements were observed in the learning speed (compare Fig 2A and 2D). Learning times are likely improved due to much more rapid development of rarely used and weak connections early in the learning process. Indeed, in this new model synaptic changes due to rewarded events may be significantly larger for the connections experiencing only rare positive STDP events. Thus implementation of synaptic trace normalization promoted competition between synapses that have very few strong STDP traces and those with many strong STDP events and eliminated bias toward synapses with higher activity. Importantly this normalization mechanism is consistent with experimental data [32, 33, 34].

#### Balancing of synaptic input: Effect of heterosynaptic plasticity

**Fig 3:**
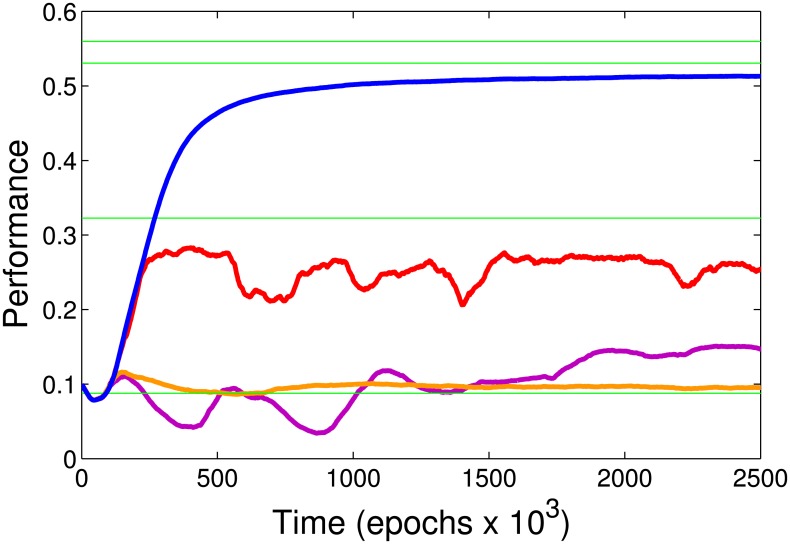
Effect of specific synaptic balancing rules on the model performance. Blue—full model with the input side heterosynaptic plasticity and output balancing. Orange—no input side heterosynaptic plasticity but output balancing is still implemented. Purple—both input side heterosynaptic plasticity and output balancing are removed. To partially compensate for the loss of the output balancing we applied another rule—the sum of all the outgoing synaptic strengths was held constant. Nevertheless, performance was further reduced to almost chance level. Red—input side heterosynaptic plasticity is implemented but no output balancing. Note the greatest impact of heterosynaptic plasticity on the model performance. Output balancing made lesser impact but still was crucial to maintaining high performance.

Next we tested the impact of the different synaptic balancing mechanisms on the model performance (Fig 3). Heterosynaptic plasticity was the most important single balancing rule [27, 28]. Its role was to ensure that the total synaptic strength of all incoming synapses to an individual neuron remains constant. Whenever a single input changed in strength, all other incoming synaptic connections of that neuron were adjusted to compensate (see Methods). As a result of maintaining a constant synaptic input, individual synapses must compete to be effective at activating the postsynaptic neuron and only those synapses which are regularly rewarded can reach sufficient strength to control the network. Without input side balancing (heterosynaptic plasticity), performance fell to near chance levels as synaptic strengths diverged to the ceiling and floor values (see Fig 3, orange line). Even when synaptic weight totals were maintained by keeping the sum of outgoing synaptic strength constant (output counter part of heterosynaptic plasticity, which would be a stronger version of the output balancing rule we discuss in the next section), performance remained at the near chance level (Fig 3, purple line) and distribution of synaptic weights became bimodal with a small number of connections reaching ceiling values.

#### Output balancing

Our previous study revealed the importance of balancing the strength of the output synapses [30]. In this new study, implementation of the output part of synaptic balancing was to reduce the rate of synaptic growth in the neurons that already had high total synaptic output. This effectively prevented a very small number of neurons from controlling the entire network. Thus, for each middle-layer cell, increments of the strength of outgoing synapses resulting from rewarded STDP events were divided by the ratio of the current sum of synaptic outputs to the initial sum of synaptic outputs of the same cells (see Methods). The result was that synapses originating from the neurons with many strong outputs were not able to increase their synaptic strength as quickly as synapses from the neurons with a weak output. This gave a competitive advantage to the later neurons. It helped to control synaptic output, thus preventing over-representation by the cells whose activities were most often correlated with the rewards (see S1 Fig). The performance of the full model simulated without this rule is shown by the red line in Fig 3.

#### Homeostatic gain control

To maintain the average desired firing rate of neurons (over long time), we implemented homeostatic scaling [20] independently for the middle and the output layer neurons. When the actual firing rate was below (above) the target rate, the efficacy of all excitatory synaptic inputs was scaled up (down) in small steps, which promoted the firing rate move towards the target. Over time this caused the firing rate of the network to gravitate around the target value. By changing the target firing rate of neurons within each layer, it was possible to control the sparseness of activity within that layer. Thus we next tested the impact of the target (homeostatic) firing rate on the model performance.

**Fig 4:**
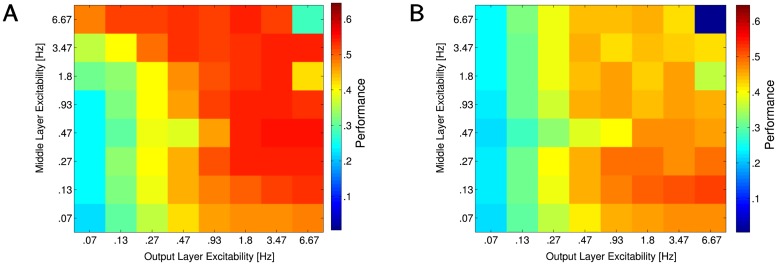
Performance plot over excitability in the middle and output network layers. A) A heat chart of peak performance under a range of target firing rates in the middle layer (y-axis) and the output layer (x-axis). B) A chart showing final performance over the same range of conditions. Points where large differences exist between the peak and the final performance charts generally indicate conditions where the network performed much better early in the simulation but suffered declines in performance over time.

For each simulation experiment, the target firing rate of neurons was set to a different level (Fig 4). We then calculated the performance for each combination of the target firing rates in two layers. When firing rates were too low, it would often prevent information to propagate down the network. Target firing rates that were too high potentially led to high peak performance but also unstable dynamics and to some form of overlearning causing reduction in performance over time. Lower firing rates and sparse activity in the middle layer were important in achieving high performance. A higher firing rate of spiking (>0.9 Hz) in the output layer revealed the best performance. This was observed because increasing mean firing rates of the output layer neurons reduced the likelihood of the spike count ties, which improved decision making. It also reduced the fraction of the movement cycles where no output spikes occurred at all (see S2 Fig in supporting information).

Effect of performance decline after reaching a maximum was likely related to an inability of the network to maintain synaptic efficacy in a moderate range. When excitability of the post synaptic cells was too high, spike events that normally only caused post synaptic spikes when occurring in coincidence with other events could eventually result in connections strong enough that even a single spike event may cause spike responses in postsynaptic neurons.

#### Role of inhibition

**Fig 5:**
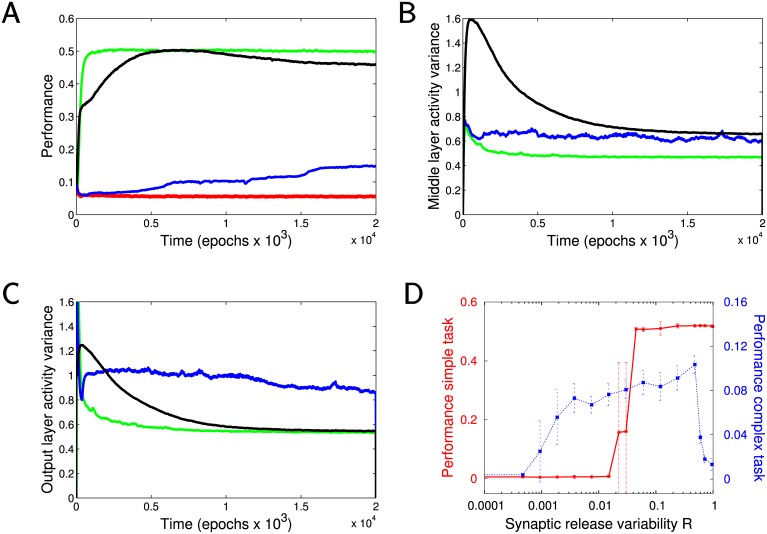
Effect of synaptic inhibition and noise. A) Model performance reduced when no inhibition was present in the network. Green—baseline (inhibition is present), black—no inhibition from input to middle layer, blue—no inhibition from middle to output layer, red—no inhibition in both layers. B) Coefficient of variance in the activity per epoch of the middle layer. C) Coefficient of variance in the output layer for the same sets of trials. Colors have the same meaning for all three panels. D) Model performance for different level of variability in synaptic release. Red—performance for the simple foraging task, blue—performance for the complex foraging task. Each dot is the average (10 independent trials) of performance measurements at the time of 2.10^4^ epochs.

Feedforward synaptic inhibition was implemented in the model (see Methods) and was necessary for optimal behavior of the network. Thus each layer projecting excitatory connections to the following layer was also projecting inhibition and the total strength of inhibition was equal to the total strength of excitation. Removing the inhibition, especially from the middle to the output layer, caused drastic reduction in performance. Fig 5A shows results of simulations in three networks with different inhibitory connectivity. The green line shows the baseline model. Removing the inhibition from the input to the middle layer revealed only a moderate decline in performance (Fig 5A, black line). When the inhibition was removed from the middle to the output layer (blue line) or there was no inhibition at all (red line), the network performed near chance level.

We found that layers not receiving inhibitory input had far greater variance in their activity per epoch (Fig 5B and 5C). Large variance in the output layer activity was associated with very poor performance. Indeed, large variance when middle->output inhibition was turned off indicates a great number of epochs with either no output activity or simultaneous spiking activity in all of the output layer neurons (see S3 Fig). Synaptic inhibition was necessary to maintain moderate levels of activity despite changes in the input strength. Thus, e.g., when many food particles were found within the network “visual field”, the activity in the input layer was high. Without inhibition at all high activity of the input layer propagated all the way to the output layer leading to increased variability between trials and great performance reduction. Eliminating inhibition only from the input to middle layers greatly increased the variance of activity in the middle layer but had far less impact on the final performance because activity in the output layer could still be constrained by feedforward inhibition to that layer.

#### Role of synaptic noise

The model included some level of randomness in all of its synaptic connections. In particular, each neurotransmitter release, following presynaptic spike, was modulated by the variable implementing a noisy component. Based on our previous work using the simplified model [30], we expected that such variability would be necessary in order for the agent to learn. Indeed, reward based learning is a trial and error process and noise allows opportunities for the correct response to occur by chance and then be rewarded and learned. Fig 5D (red line) shows that the low noise amplitude prevented the model from learning successfully, increasing the level of noise beyond a certain level led to an abrupt performance increase. When similar analysis was applied to the complex foraging task, as described in the next section, we found a smoother transition (Fig 5D, blue line). Nevertheless, a certain level of noise was still required for our model to learn successfully.

### Complex foraging behavior: Pattern classification and learning

**Fig 6:**
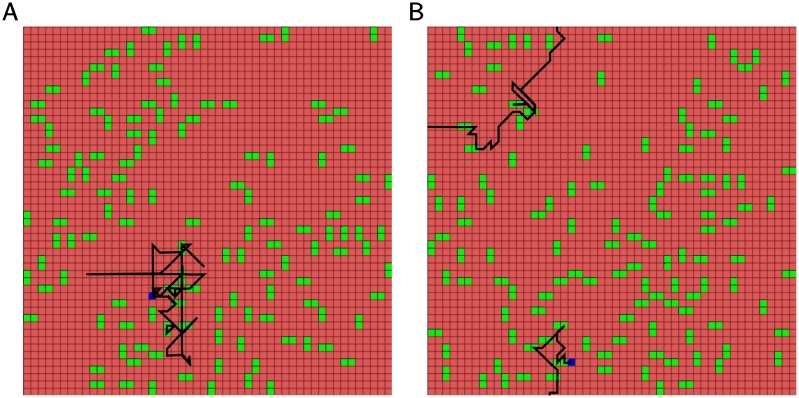
Environment arrangement and agent behavior. Green particles represent food; horizontal patterns are rewarded, vertical patterns are punished. Blue dot is a starting point of the agent. Black line represents its movement in sequential steps. A) Moving behavior before learning. B) Moving behavior after learning. Note the avoidance of vertical patterns.

Our previous model with only one plastic layer was able to solve a simple foraging task of food acquisition at any location regardless of the food pattern shape [30]. It failed, however, to distinguish between patterns of different shape and to acquire only food particles arranged in a particular way. In contrast, the new model with two plastic layers, was able to accomplish this more complex task. In the following experiments the food in the environment was arranged into vertical and horizontal pairs of locations. When the virtual agent arrived at the location of either particle from a pair, both particles were removed and another pair of food was generated at a random location on the map. Obtaining horizontally arranged food was rewarded, while obtaining vertically arranged food was punished. Thus, vertically arranged food was considered to be “bad” or “toxic”. When food particles were placed in the environment, it was arranged in such a way that one pattern was never adjacent to another to avoid ambiguity of the input. The two layer model was able to quickly learn avoiding vertical food arrangements and only acquire food particles arranged horizontally (see Fig 6).

#### Complementary roles of rewarded and non-rewarded STDP

Simple pattern classification and approach/avoidance behavior was achieved in our model through a combination of rewarded and non-rewarded STDP. Inputs to the middle layer were affected by non-reward modulated STDP (see Methods). The upper limit on the strength of synaptic connections from the input to the middle layer neurons was such that no single synaptic input was sufficient to evoke a spike in a middle layer cell. Inhibitory connections from the input to the middle layer were shown to have a minimal impact on the base model performance (Fig 5A, black line) and we did not include inhibition in the pattern-classifying network because it reduced the reliability for a pair of inputs to evoke a response in the middle layer.

**Fig 7:**
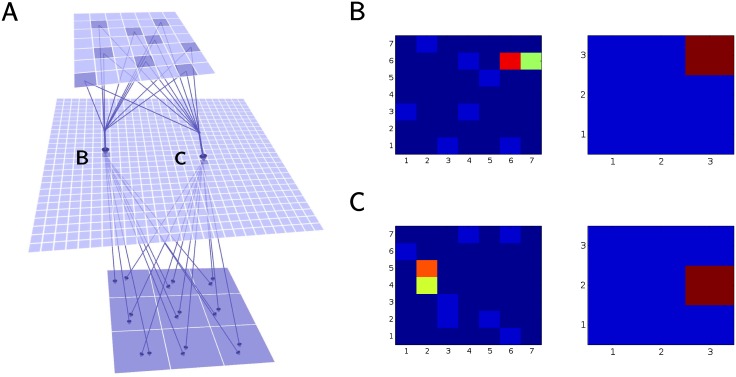
Structure of synaptic connectivity after learning. A) Network connectivity diagram for two typical middle layer cells. On top is input layer (visual field), below is the middle layer network and bottom is the output layer (direction of next movement). B&C) Strengths of the synaptic inputs (left) and outputs (right) of two typical middle layer neurons (labeled in panel A as ‘B’,‘C’), after successful training. The network has been trained to move toward horizontal food pairs and away from vertical food pairs. Red represents the highest synaptic strength while blue represents the lowest. B) A characteristic middle layer cell that has become responsive to a pair of horizontally arranged inputs on the top right of the visual field and excites the top-right output cell that directs movement of the virtual agent toward the food pair. C) Another characteristic middle layer cell that has become responsive to a pair of vertically arranged inputs on the left of the visual field. It has learned to excite the right-direction cell that directs movement away from food pair.

Over time, middle layer neurons learned to respond only when a specific pair of input layer neurons fired together. More commonly co-occurring pairs of events developed significantly more representation in the middle layer. As food particles were presented as horizontal or vertical pairs in the environment, most middle layer neurons became responsive randomly to either a vertical or a horizontal pair of cells in the input layer (see Fig 7), thus giving an example of a basic generalization behavior while still maintaining location based specificity [35]. The connectivity pattern from the input layer to the middle layer impacted both the model performance and its computational complexity. We found that the optimal compromise between the learning and the computational performance was reached with fan-in about 9 input neurons (see S4 Fig) and this was used in the rest of the study. Interestingly, for complex tasks increase of connectivity beyond this limit led to some decrease of performance.

The output layer received all to all connections from the middle layer. Recent STDP traces were rewarded when the virtual agent acquired horizontally arranged food pairs and were punished when the agent acquired vertically arranged food pairs. Thus,the network learned to move toward horizontal (rewarded) pairs and to avoid the vertical (punished) pairs over the course of training.

Characteristic structure of synaptic connectivity after training was formed for the simpler version of a foraging task, where for a given middle layer cell the input from specific input layer cell would become prominent and its output increased to the corresponding quadrant of the “decision” layer, pursuing approaching behavior (see S5 Fig in supplementary information).

#### Performance

**Fig 8:**
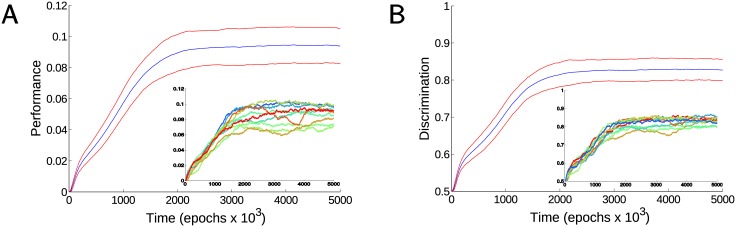
Model performance for complex discrimination task. A) Performance for a task requiring the discrimination of stimuli between vertical and horizontal food pairs. Blue line represents the mean performance and red lines represent the mean ± the standard deviation. Note that asymptotic performance was lower than that for the base model (e.g., Fig 2A) because of the lower overall food density (see Methods) and avoidance of the “bad” food. Inset: The performance of 10 typical trials. B) Average rate of rewarded (“good”) food acquired as a percentage of the total rate of food acquired for the same set of trials as in A. Inset shows the same measure for 10 sample trials.

Fig 8A shows performance of the model for a complex discrimination task. In order to let enough “freedom of movement” to the virtual agent, the total density of rewarded food particles in the new environment was 25% compared to the base model where no compound shapes were used (see Fig 2). This implies that the value of the optimal performance of an ideal agent for complex discrimination task should be approximately one fourth of that in the base model (performance is defined as a rate of obtained food). Accuracy in discriminating between the two food arrangements was often above 80% with high rate of food acquisition (Fig 8B). When high performance levels were obtained, the network developed in such a way that the majority of middle layer cells had most of their synaptic inputs originating from different pairs of input layer cells (see Fig 7B and 7C).

Our initial analysis revealed that for a fraction of trials after a short period of learning performance could drop to zero and then it would stay very low. We found that for reliable learning and avoiding this problem, the punishment associated with “bad” food (vertical pattern) had to be lower than the reward associated with acquisition of “good” (horizontal pattern) food. When the net sum of reward and punishment was near zero, the “crash” in performance, as described above, could often follow. When the magnitude of reward was set to be twice as large as punishment, a reliable learning with comparable learning times was observed in all trials.

## Discussion

We applied a neuronal network of spiking neurons with synaptic plasticity to model a simple foraging task where the virtual agent was required to navigate toward areas of high food concentration. Previously we found that a model with a single plastic layer was able to accomplish this task [30]. In this new study the network employing two plastic layers, combining un-rewarded (unsupervised learning) and rewarded (reinforcement learning) spike time dependent plasticity (STDP) was capable of pattern discrimination and also achieved higher performance.

The model simulated a virtual agent moving through a 50 by 50 grid world with “food” participles randomly distributed throughout it. An input layer of the network (7 by 7) received excitation to the neurons representing locations of the food particles in the “visual field”; an output layer (3 by 3) controlled the virtual agent direction of movement. The middle (hidden) layer received plastic (STDP) connections from the input layer and projected plastic (rewarded STDP) connections to the output layer. Multiple homeostatic mechanisms were implemented to maintain synaptic homeostasis. No labeled training data set was necessary or implemented. Over the course of continuous learning, the network developed the ability to move toward concentrations of food and learned to discriminate between “good” food and “bad” food based on the spatial arrangement of the food particles. The noise in the system promoted trial-and-error learning and helped to select new efficient problem solving strategies. We found that a combination of rewarded and non-rewarded STDP within multiple layers of plasticity was necessary to successfully perform this complex foraging task.

### Using STDP as a plasticity rule

While STDP has been previously applied in the network models to explain pattern recognition [36], and rewarded STDP has been shown to be capable of overcoming the distal reward problem [12, 37, 38, 39, 16, 17], our study combined both types of synaptic plasticity in a single model to achieve high learning performance in discrimination and decision making tasks. By punishing the network when incorrect decision (wrong type of food is acquired) was made, the network was required (a) to identify the input pattern (food type) by recognizing the arrangement of the food participles and (b) to learn to move toward the rewarded pattern (horizontal arrangement of food particles) and away from the punished pattern (vertical arrangement of food particles).

It has been shown that STDP is most relevant to cells firing in the mid range frequencies, while other rules, depending primarily on the presynaptic firing rate, may become important for the networks operating in the high frequency range [40, 41, 42]. Combining both rate-based and time-based plasticities gives rise to more complex network connectivity patterns [43, 44] which could potentially be used for more complex tasks. Our model does not employ the rate-based plasticity but future extensions of our model may incorporate a combination of these complementary forms of synaptic learning.

### Synaptic balancing

The idea of synaptic strength normalization was formulated shortly after Hebb’s seminal work [45]. It was shown that for a successful Hebbian cell assembly to develop, the total sum of input synaptic weights has to be held near constant in order to prevent runaway dynamics [46, 47]. This leads to a strong need for extensive homeostatic regulation to prevent pathological synchronized activity and indeed there is evidence that homeostatic mechanisms are deeply involved in both preventing and causing epileptic conditions [48, 49, 50, 51]. Such normalization rules became an integral part of modeling studies of supervised learning and cortical map formation [52].

In our model, maintaining the balance of synaptic strength within the network was vital to learning and to achieving high model performance. However, not all synaptic rules contributed equally to the final performance of the model. By far the most important was heterosynaptic plasticity which reduces (increases) the strength of all other synapses when one synapse increases (reduces) in strength by homosynaptic plasticity. Such a tendency towards maintaining the net input to a neuron has already been found in experimental studies [53, 54, 26, 27] and has been tested in detailed biophysical models [25, 28]. Its potential mechanism may depend on the intracellular Ca^2+^ dynamics which is influenced by the backpropagating action potentials [55, 56] at the synapses which do not actively participate in homosynaptic plasticity induction [24, 23, 22, 57, 21].

We also found that the model could only operate efficiently when the net network activity remained within a certain range. If the network became overactive, high levels of activity in the output layer made direction selectivity highly inconsistent. If the network activity was too low, not enough input would be received by the output layer to cause spiking, leading to poor performance as well. Control of the long term average network activity can be achieved by homeostatic scaling [58, 59], which adjusted the strength of synaptic inputs up for the neurons with low average firing rates and down for the neurons with high average activity. Whenever there was variation in food density near the agent, it was important to maintain comparable firing rates in the output neurons. Keeping a functional range of this activity from one trial to the next required that the network activation did not increase in direct proportion to the input activity.

As the highly active and more consistently rewarded synapses regularly increased in strength, it was possible for certain neurons to gain a major control over the network dynamics. Although it was not necessary that all neurons be equally represented, having a small number of neurons exert much greater control over the network had a pronounced negative effect on performance. Output-side synaptic balancing allowed us to reduce inequalities in the representation of the neurons and created competition between the outputs of a neuron as was proposed in [30]. This was particularly useful when a middle-layer neuron had two (or more) strong connections to the output neurons, thus “supporting” two different directions of movement. If such middle-layer neuron received a reward, it would increase strength of both connections—despite the fact that only one of them was actually responsible for correct food acquisition. Output-side balancing allowed only synapses correlated with reward to be strengthened via rewarded STDP traces. While we do not know direct experimental evidence for this mechanism, a similar effect can be potentially achieved by more complex circuits involving reciprocal inhibition between output neurons.

We observed that by normalizing the amount of potentiaton in STDP (based on the history of STDP traces occurring in the synapse past), we increased the speed of initial learning and increased the stability of efficiency gains for the network in the long term. Though not directly observed, this mechanism is consistent with experimental data [32, 33, 34] and might be of interest for additional experimental scrutiny.

### Inhibition

Interaction between synaptic excitation and inhibition is a ubiquitous phenomenon found across many different brain circuits [60, 61, 62, 63, 64, 65, 66, 67]; it can be observed during spontaneous activity of the brain [68, 69, 70] and is an integral part of the oscillatory mechanisms in the neuronal networks [71]. To simplify implementation of the inhibitory effects in our model, the neurons of the preceding layer have sent inhibitory connections to the same set of post synaptic cells they excited in the following layer. This essentially provided feedforward inhibition between layers of neurons, using a population of virtual inhibitory neurons that was not explicitly modeled. The total strength of inhibitory connections from any given cell was the same as the total of its excitatory connections. While not biologically realistic, this simplification effectively simulated a feed forward inhibition found in various brain areas [72] where neurons in one layer project to both excitatory and inhibitory neurons in the down stream layer and inhibitory neurons in turn project to excitatory neurons in the same layer [73]. As inhibitory neurons are often more electrically compact [74] and have a faster membrane time constant, this can result in the inhibition arriving to the post synaptic excitatory neurons with only a small delay compared to the excitation. No significant difference was seen in performance of this network as a result of this simplified implementation of inhibition when compared to the previous model where inhibition was implemented explicitly [30].

### Noise

Noise is another ubiquitous phenomenon in the neural networks and spans multiple time scales and different physical domains [75]. In contrast with artificially crafted devices where noise is usually detrimental to functioning, biological neural networks are capable of using noise in a productive way to improve efficiency and performance [76, 77]. Examples of this include enhanced input detection by means of stochastic resonance [78, 79, 80, 81] and probabilistic inference [82, 83]; noise can help with performance of the associative memories [84], smoothing the threshold for action potentials [85, 86], allowing rapid response time for neuronal populations [87, 88] or faithful propagation of firing rates across a layered network [89].

The largest source of the noise in any biological neuronal network is synaptic noise [90]. In our model we employed noise at the level of synaptic currents that represented variability in synaptic vesicle release. Noise was not only tolerated by this model but it was required for its function. Before training there was no meaningful mapping between the input received by the network and its output; the output activity (and therefore direction of movement) was essentially driven by random initial configuration of synaptic weights. Variability (noise) in synaptic release occasionally caused an unexpected output for a given input. When the network output resulted in food acquisition, it was reinforced by a reward. If a synapse that was likely to be associated with a reward was able to surpass the strength of another synapse of the same presynaptic cell, noise in the synaptic output created instances where the “correct” output cell fired and the “incorrect” output cell did not, despite the “incorrect” output cell having a stronger incoming synaptic strength. This effectively allowed for a form of trial and error learning. Without such variety of the actions attempted, it would be difficult to find which goal-oriented strategies are effective and it can be seen as representing a strategy of associating a given stimulus with a given response.

### Conclusions

Unlike common machine learning approaches or the currently booming deep network architectures [91] which, in some cases, have already outperformed humans [92, 93, 94, 95, 96], in this study we only applied biologically plausible and experimentally identified plasticity rules. This new study, following our previous work [30], generated specific predictions regarding what biological learning mechanisms are necessary and/or sufficient to accomplish the learning task. The key components for a successfully working model were STDP, synaptic balancing processes keeping the learning stable, dopamine type reward feedback derived from successful outcome of action and noise at the synaptic level allowing trial-and-error learning. Although these components have already been studied, sometimes in great detail, our study links them together (unsupervised STDP for sensory learning and rewarded STDP for decision making in particular) in a new way and shows advantages of interaction between different plasticity mechanisms for complex task solving.

## Methods

### Environment

Foraging behavior took place in a virtual environment of randomly distributed “food” particles. The environment consists of 50 by 50 grid of locations. Initially, each location was either assigned or not a food particle. When a food particle was acquired as a result of the virtual agent move, it was then assigned randomly to a new spot on the map. This resulted in a continuously changing environment with the same food density. The density of food particles in the environment was set to 10%. The virtual agent was seeing a 7 by 7 grid of squares the (“visual field”) centered on its current location and it could move to any adjacent square including diagonally for a total of 8 directions.

### Network structure

The network was composed of 842 spiking map based neurons [97, 98], arranged into 3 feed forward layers to mimic a basic biological circuit: a 7 by 7 input layer (I), a 28 by 28 middle (hidden, H) layer, and a 3 by 3 output layer (O) (Fig 1). This structure provides a basic feedforward inhibitory circuit [73] found in many biological structures [99, 73, 100, 101, 102, 103].

Each cell in the middle layer received synaptic inputs from 9 random cells in the input layer. These connections initially had random strengths drawn from a normal distribution. Each cell in the excitatory middle layer (cell *H*_*i*_) connected to every cell in the output layer (*O*_*j*_) with synaptic strength *W*_*ij*_ or *WI*_*ij*_, respectively. Initially all these connections had uniform strengths and the responses in the output layer were due to the random synaptic variability. Random variability was a property of all synaptic interactions between neurons and it was implemented as variability in the magnitude of the individual synaptic events.

### Movement cycle

Simulation time was divided up into epochs of 600 time steps, each roughly equivalent to 300 ms. At the start of each epoch the virtual agent received input corresponding to locations of nearby food within the input (7x7) layer. Thus 48 of the 49 cells receive input from a unique position relative to the virtual agent location. At the end of the epoch the virtual agent made one move based on the activity of the output layer. If the virtual agent moved to a grid square with “food”, the “food” was removed from that square and assigned to a randomly selected new square.

Each epoch was of sufficient duration for the network to receive inputs, produce outputs, and return to a resting state. Input layer neurons representing positions in the environment containing food received brief pulse of excitatory stimulation that triggered a spike; this stimulation was evoked at the start of each movement cycle (epoch). Output was chosen and the virtual agent moved at the end of the epoch.

The activity of the output layer of the network controlled direction of virtual agent’s movement. Each of the output layer cells was mapped to a specific direction. The output layer cell (*O*_*j*_) that spiked the greatest number of times during the first half of an epoch defined the direction of movement on that epoch. If there was a tie, direction was chosen randomly from the tied outputs. If no cells in the output layer fired, the virtual agent continued in the direction it traveled during the previous epoch.

There was 1% chance on every move that the virtual agent would ignore any output and instead move in a random direction. This random variability prevented infinite loops of virtual agent’s motion during the learning process. Synaptic noise was not sufficient to break out of all movement loops as some loops were the result of forming strong connections that would mediate the same spiking pattern regardless of the noise. Other times the probability of escape from a loop due to the noise was simply low enough that it would take a significant amount of time to break the loop. While biological systems could utilize different mechanisms to achieve the same goal, the method we implemented was efficient and accomplished the goal.

In the model where no pattern recognition was required, the chance of random movement started at 0.5% but increased by 0.5% for each move in which no food was obtained. This value was reset to its starting value whenever food was obtained.

For more complex task where pattern recognition was also required, high performing solutions to the task included both approach and avoidance behavior. As such, the network was far more susceptible to becoming stuck in a movement cycle, usually when the virtual agent became surrounded by “toxic” food. In order to break these cycles the rate of random motion was gradually increased by 1% per move in which the agent did not obtain food.

### Synaptic plasticity

Synaptic plasticity closely followed the rules introduced in [30]. A rewarded STDP paradigm [12, 37, 38, 104] was implemented between layers H and O and a non-reward modulated STDP paradigm between I and H. A spike in a post-synaptic cell (*O*_*j*_ of the output layer) that directly followed a spike in a pre-synaptic cell (*H*_*i*_ of the hidden layer) created a “*pre before post*” event. Each new post synaptic cell spike was compared to all pre synaptic spikes within the time window and each new pre synaptic spike was compared to all postsynaptic spikes within the window.

The value of an STDP event (trace) was calculated using the following equation [10, 105]:

$$\begin{array}{ccc} p & = & \frac{-|t_{r}-t_{p}|}{T_{c}}\;, \end{array}$$

$$\begin{array}{ccc} tr_{k} & = & Ke^{p} \end{array}$$ (1)

where *t*_*r*_ and *t*_*p*_ are the times at which the pre and post synaptic spiking events occurred respectively, *T*_*c*_ is the time constant and is equal to 40 ms. *K* is equal to —0.04 in the case of a *post before pre* event and 0.04 in the case of a *pre before post* event.

STDP event was immediately applied to the respective synapse *W*_*ij*_ between neurons *I*_*i*_ and *H*_*j*_. In contrast, for synapses between neurons *H*_*i*_ and *O*_*j*_ the events were stored as traces for later use. Each trace remained stored for 6 epochs after its creation and then was erased. While still stored, STDP trace had an effect whenever there was a rewarding or punishing event. If the network was rewarded or punished the new synaptic strength of the synapse *W*_*ij*_ was described as:

$$\begin{array}{ccc} W_{ij}\left(n+1\right) & = & W_{ij}\left(n\right)\prod_{k}^{traces}(1+\frac{W_{i0}}{W_{i}}*\Delta_{k})\;, \end{array}$$ (2)

$$\begin{array}{ccc} \Delta_{k} & = & S_{rp}\cdot \frac{tr_{k}}{t-t_{k}+c}\cdot \frac{Sum_{tr}\left(n+1\right)}{Avg_{tr}\left(n+1\right)}\;, \end{array}$$

$$\begin{array}{ccc} Sum_{tr}\left(n+1\right) & = & \sum_{k}^{traces}\frac{tr_{k}}{t-t_{k}+c}\;, \end{array}$$

$$\begin{array}{ccc} Avg_{tr}\left(n+1\right) & = & Avg_{tr}\left(n\right)\left(1-\delta \right)+\delta Sum_{tr}\left(n+1\right)\;, \end{array}$$

where *t* is current time step, *S*_*rp*_ is a scaling factor for reward/punishment, *tr*_*k*_ is magnitude of trace (defined in Eq (2)), *t*_*k*_ is time of the trace event, *c* is a constant (=1 epoch) used for decreasing sensitivity to very recent spikes, *W*_*i*_ = ∑_*j*_ *W*_*ij*_ is a total synaptic strength of all connections from specific cell *H*_*i*_ to all cells *O*_*j*_ of the output layer, *W*_*i*0_ is a constant that is set to the value of *W*_*i*_ at the at the beginning of the simulation (“target value”). The term *W*_*i*0_/*W*_*i*_ helped to keep the output weight sum close to the initial target value. The values for *Avg*_*tr*_ and *Sum*_*tr*_ were almost always positive in our simulations due to the feed forward architecture that we used. We should note, that for a more general model with feedback loops it would be more appropriate to handle negative and positive traces separately.

The network was rewarded when the virtual agent moved to a “food” location and *S*_*rp*_ = 1. In case of pattern recognition model it was punished when it moved to a location with a toxic food and *S*_*rp*_ = −0.5. There was also smaller punishment applied when no food is obtained, *S*_*rp*_ = −0.1 in the base model and *S*_*rp*_ = −0.01 in the pattern recognition model. The effect of these rules was that the cells with lower total output strength increased their output strength more easily.

To ensure that all the output neurons maintained a relatively constant long term firing rate, the model incorporated homeostatic synaptic scaling, which takes place every epoch (= 600 time steps). The total strength of synaptic inputs *W*_*j*_ = ∑_*i*_ *W*_*ij*_ to a given output cell *O*_*j*_ was set to be equal at each time step to the target synaptic input *W*_*j*_ = *W*_*j*0_—a slow variable that varied over many epochs and depended on the activity of the cell *O*_*j*_ and activity of its all pre-synaptic cells. If a cell *O*_*j*_ consistently fired below the target rate, the *W*_*j*0_ was increased by *D*_*tar*_ = 0.0001. If the cell responded above its target firing rate the *W*_*j*0_ was gradually reduced:

$$\begin{array}{ccc} W_{j\left(n+1\right)} & = & \{\begin{array}{cc} W_{j\left(n\right)}*\left(1+D_{tar}\right) & \text{spike}\;\text{rate}<\text{target}\;\text{rate}\\ W_{j\left(n\right)}*\left(1-D_{tar}\right) & \text{spike}\;\text{rate}>\text{target}\;\text{rate} \end{array} \end{array}$$ (3)

To ensure that the net synaptic input *W*_*j*_ to any neuron was unaffected by plasticity events of the individual connections at the individual time steps and equal to *W*_*j*0_, we implemented scaling process that occurs after each STDP event. When any excitatory connection increased in strength, all the other excitatory connections incoming to that cell decreased in strength by a “*scale factor*” *S*_*f*_ to keep *W*_*j*_ = *W*_*j*0_:

$$\begin{array}{ccc} W{}_{ij(n+1)} & = & W_{ijn}S_{f} \end{array}$$

$$\begin{array}{ccc} S_{f} & = & \frac{W_{j0}}{\sum_{i}W_{ijn}} \end{array}$$ (4)

*W*_*ijn*_ are synaptic weights right after STDP event but before scaling, *W*_*ij*(*n*+1)_ are synaptic weights after scaling, *W*_*j*0_ is *W*_*i*0_ from Eq (2).

### Map based neuronal models

The underlying reduced model of fast spiking neuron was identical to the model used in [30] and can be described by the following set of difference equations [97, 106, 107]:

$$\begin{array}{c} V_{n+1}=f_{\alpha }\left(V_{n},I_{n}+\beta_{n}\right)\;, \end{array}$$ (5)

$$\begin{array}{c} I_{n+1}=I_{n}-\mu \left(V_{n}+1\right)+\mu \sigma +\mu \sigma_{n\;,} \end{array}$$

where *V*_*n*_ is the membrane voltage, *I*_*n*_ is a slow dynamical variable describing the effects of slow conductances, and *n* is a discrete time step (~0.5 msec). Slow temporal evolution of *I*_*n*_ was achieved by using small values of the parameter *μ* ≪ 1. Input variables *β*_*n*_ and *σ*_*n*_ were used to incorporate external current $I_{n}^{ext}$ (e.g., synaptic input): $\beta_{n}=\beta^{e}I_{n}^{ext}$, $\sigma_{n}=\sigma^{e}I_{n}^{ext}$. Parameter values were set to *σ* = 0.06, *β*^*e*^ = 0.133, *σ*^*e*^ = 1, *μ* = 0.0005. The nonlinearity *f*_*α*_(*V*, *I*) was designed in the form of a piece-wise continuous function:

$$\begin{array}{c} f_{\alpha }\left(V_{n,I_{n}}\right)=\{\begin{array}{cc} \alpha {\left(1-V_{n}\right)}^{-1}+I_{n}, & V_{n}\leq 0\\ \alpha +I_{n}, & 0<V_{n}<\alpha +I_{n}\;\&\;V_{n-1}\leq 0\\ -1, & \alpha +I_{n}\leq V_{n}\;\text{or}\;V_{n-1}>0\;, \end{array} \end{array}$$ (6)

where *α* = 3.65. To convert the dimensionless “membrane potential” *V* to the physiological membrane potential *V*_*ph*_, the following equation was applied: *V*_*ph*_ = 50*V* − 15 [mV] [98].

This model is very computationally efficient and, despite its intrinsic low dimensionality, produces a rich repertoire of dynamics capable to mimic the dynamics of the Hodgkin-Huxley type neurons both at the single cell level and in the context of network dynamics [97, 107]. A fast spiking neuron model was chosen to simulate the neurons.

To model synaptic interconnections, we used conventional first order kinetic models of synaptic conductances rewritten in the form of difference equations:

$$\begin{array}{c} g_{\left(n+1\right)}^{syn}=\gamma g_{n}^{syn}+\{\begin{array}{cc} \left(1+XR\right)g_{syn}/W_{j}, & spike_{pre},\\ 0, & otherwise, \end{array} \end{array}$$ (7)

and the synaptic current computed as: $I_{n}^{syn}=-g_{n}^{syn}(V_{n}^{post}-V_{rp})$.

Here *g*_*syn*_ is the strength of synaptic coupling, modulated by the target rate *W*_*j*_ of receiving cell *j*, indices *pre* and *post* stand for the presynaptic and postsynaptic variables, respectively. The first condition, “*spike*_*pre*_”, is satisfied when presynaptic spikes are generated. Parameter *γ* controls the relaxation rate of synaptic conductance after a presynaptic spike is received (0 ≤ *γ* < 1). The parameter *R* is the coefficient of variability in synaptic release. The standard value of *R* is 0.12. X is a randomly generated number between -1 and 1. Parameter *V*_*rp*_ defines the reversal potential and, therefore, the type of synapse: excitatory or inhibitory. The term (1+XR) introduces a variability in synaptic release such that the effect of any synaptic interaction has an amplitude that is pulled from a flat distribution ranging from 1+R to 1-R times the average value of the synapse.

## Supporting information

S1 Fig Average synaptic strengths of the input layer neurons. Input layer neurons (“visual field”) were divided into 3 groups based on the distance from the center (agent position). For each group, we measured the average total strength of the connections to the middle layer cells. Left: baseline model; Right: output balancing disabled. X-axis is a distance from the center. Y-axis is the average connection strength, averaged over 20 independent trials. Output balancing helped to keep the average synaptic strengths for all three groups of neurons in the same range, while the network without output balancing developed large differences between the groups. Even distribution of the outputs helped the model to learn equally the information about distant and nearby food, yielding better results in the overall performance as shown in the Fig 3.
(PDF)

S2 FigEffect of the output layer neurons’ excitability on the outcome of the decision making.Left: number of non-zero ties between output neurons, middle: number of the epochs with no response of the output neurons (all cells in the output layer remained silent during epoch), right: both ties and zeros counted together. Each dot is average of 10 independent trials. The mean output layer firing rate was considered as a measure of excitability; $1.\bar{6}$ Hz was the default output firing rate. Note, that the number of non-zero ties was small and the number of epochs with no response was high for the low excitability (< 1 Hz), because the excitability was too low to reach the spiking threshold and the output layer commonly remained silent. Decreasing number of non-zero ties for high excitability (>10Hz) was observed because the likelihood of exact tight became low as the number of spikes generated by the output neurons increased.(PDF)

S3 Fig Effect of inhibition on the output layer activity. Left, the histogram shows baseline activity (inhibition enabled). X-axis gives total number of spikes in the output layer during single epochs; Y-axis—number of epochs for each class of firing (distribution). Next two histograms show the network activity when no input->middle layer or no middle->output inhibition, respectively, was implemented. All data are from 10 independent trials for each scenario.
(PDF)

S4 Fig The role of fan-in to the middle layer. Performance of the model with respect to the varying fan-in from the input to the middle layer cells. Each point is a final performance averaged from 10 independent trials, each trial running for 2.10^4^ epochs. Left: Simple task. From fan-in > 4, the model reached performance close to the optimum level. Right: Complex task. The learning performance was almost zero for fan-in < 4, gradually improving until it peaked around 8-9 and then decreasing slowly for even higher fan-in connectivity.
(PDF)

S5 Fig Structure of synaptic connectivity after learning a simple foraging task. Strengths of the synaptic inputs (left) and outputs (right) of a typical middle layer neuron after successful training. The network has been trained to move toward any (single) food particle. Red represents the highest synaptic strength while blue represents the lowest strength. A characteristic middle layer cell that became responsive to a single food particle in the top right of the visual field (left) and excited the top right output cell (right) which moved the virtual agent toward the food particle.
(PDF)
