# Supplementary figures and images for: Multi-layer network utilizing rewarded spike time dependent plasticity to learn a foraging task
Source: PLoS Comput Biol. 2017 Sep 29;13(9):e1005705. doi: 10.1371/journal.pcbi.1005705 (PMC5636167; doi:10.1371/journal.pcbi.1005705)

Baseline

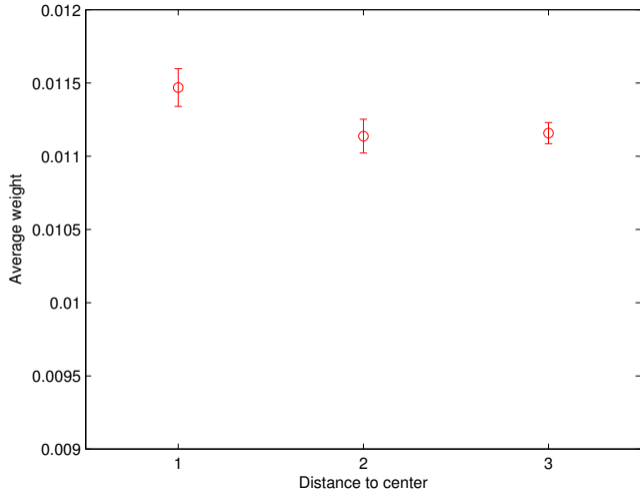

Output balancing disabled

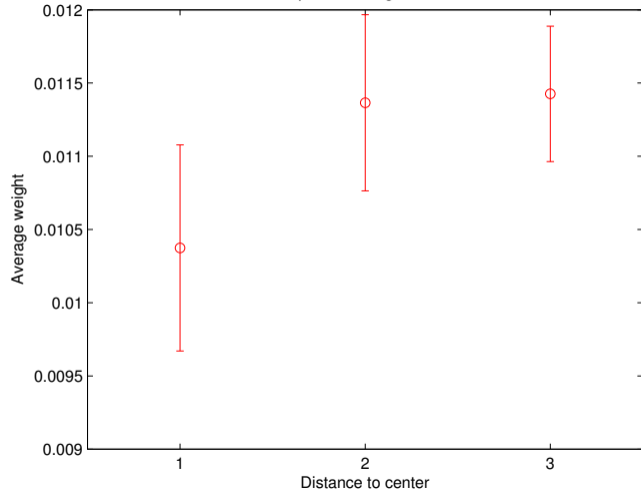

Supplement: S1 Fig — Input layer neurons (“visual field”) were divided into 3 groups based on the distance from the center (agent position). For each group, we measured the average total strength of the connections to the middle layer cells. Left: baseline model; Right: output balancing disabled. X-axis is a distance from the center. Y-axis is the average connection strength, averaged over 20 independent trials. Output balancing helped to keep the average synaptic strengths for all three groups of neurons in the same range, while the network without output balancing developed large differences between the groups. Even distribution of the outputs helped the model to learn equally the information about distant and nearby food, yielding better results in the overall performance as shown in the Fig 3. (PDF) [file pcbi.1005705.s001.pdf]

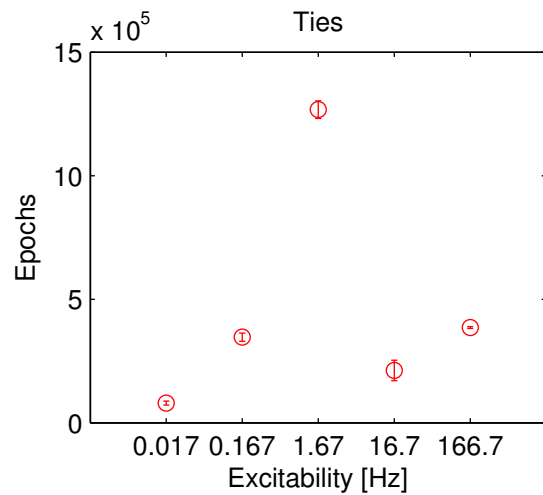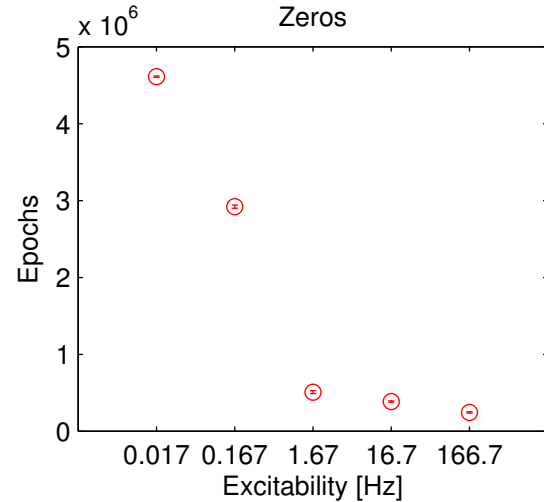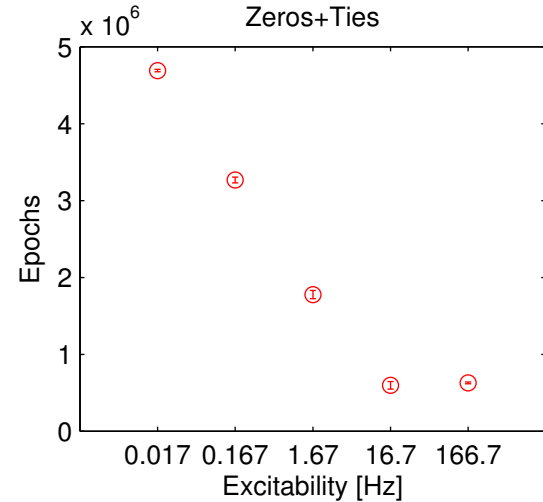

Supplement: S2 Fig — Left: number of non-zero ties between output neurons, middle: number of the epochs with no response of the output neurons (all cells in the output layer remained silent during epoch), right: both ties and zeros counted together. Each dot is average of 10 independent trials. The mean output layer firing rate was considered as a measure of excitability; 1.6‾ Hz was the default output firing rate. Note, that the number of non-zero ties was small and the number of epochs with no response was high for the low excitability (< 1 Hz), because the excitability was too low to reach the spiking threshold and the output layer commonly remained silent. Decreasing number of non-zero ties for high excitability (>10Hz) was observed because the likelihood of exact tight became low as the number of spikes generated by the output neurons increased. (PDF) [file pcbi.1005705.s002.pdf]

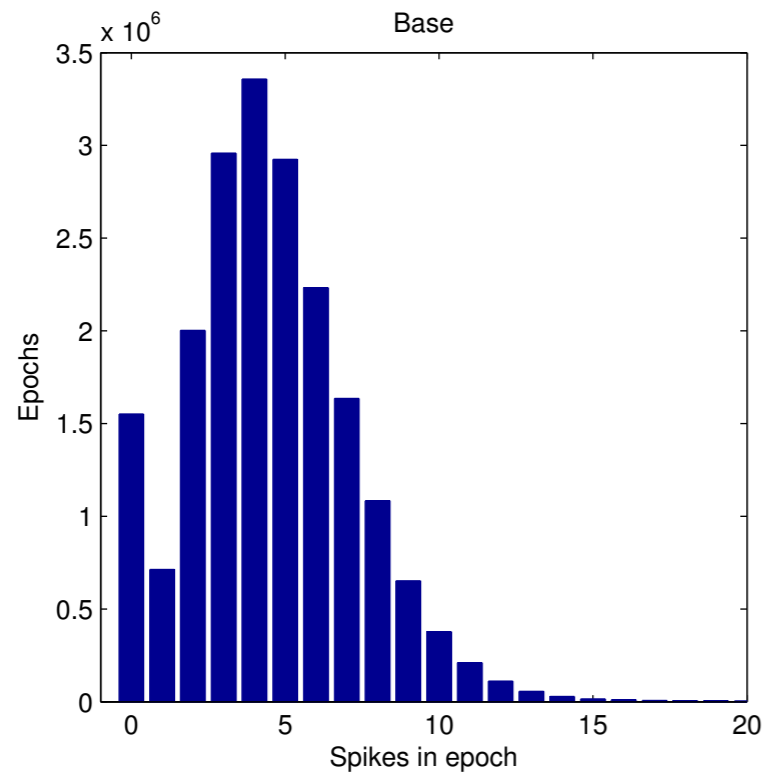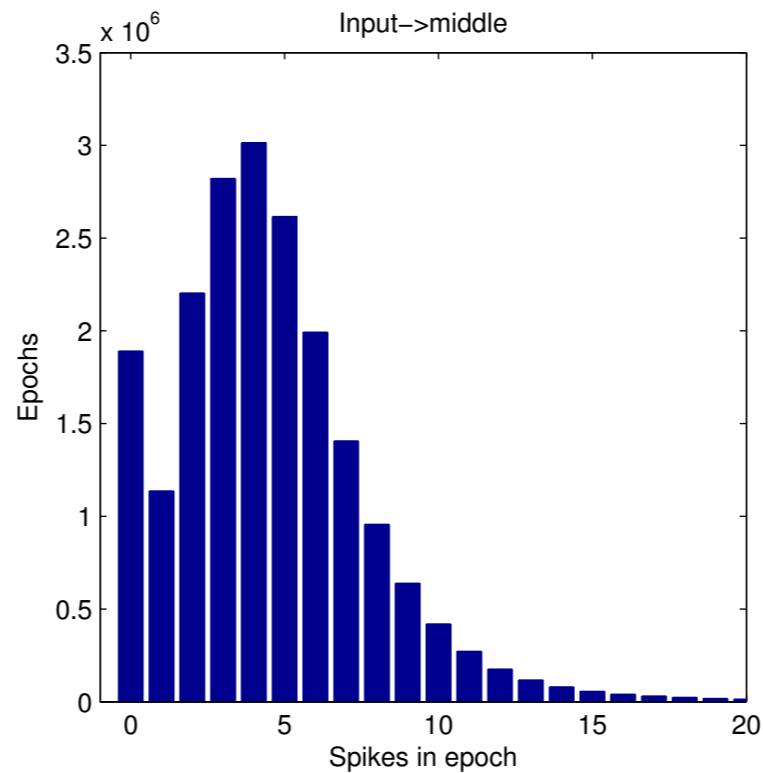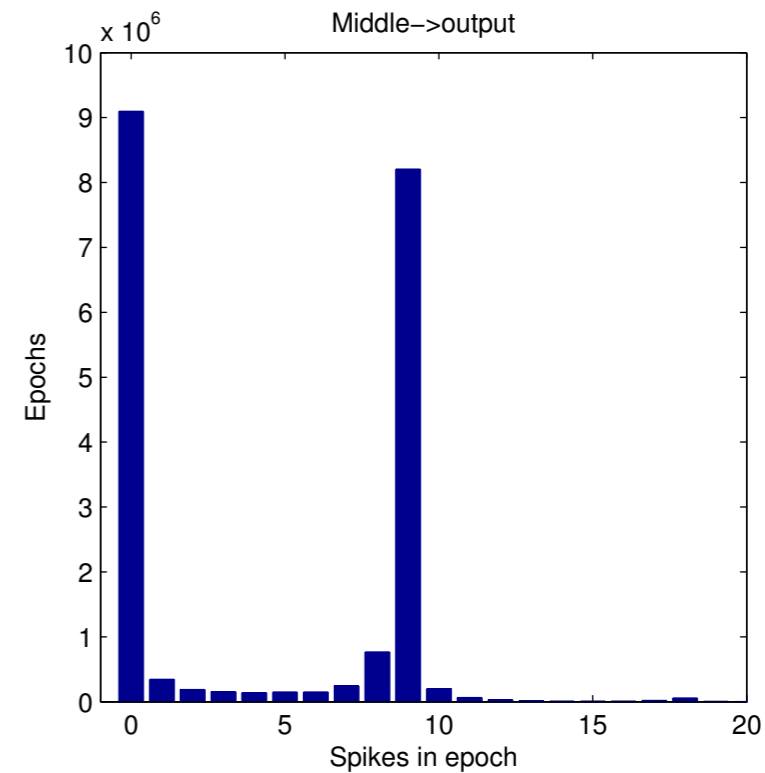

Supplement: S3 Fig — Left, the histogram shows baseline activity (inhibition enabled). X-axis gives total number of spikes in the output layer during single epochs; Y-axis—number of epochs for each class of firing (distribution). Next two histograms show the network activity when no input->middle layer or no middle->output inhibition, respectively, was implemented. All data are from 10 independent trials for each scenario. (PDF) [file pcbi.1005705.s003.pdf]

Avg. performance

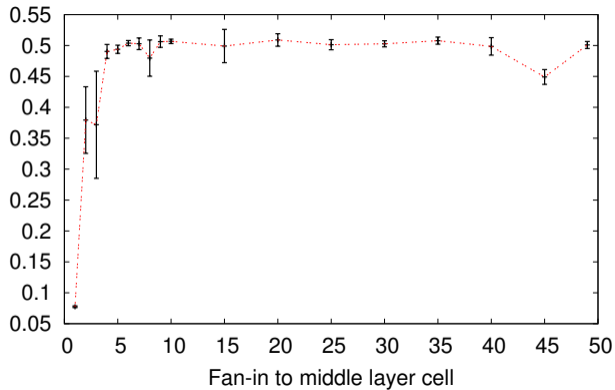

Avg. performance

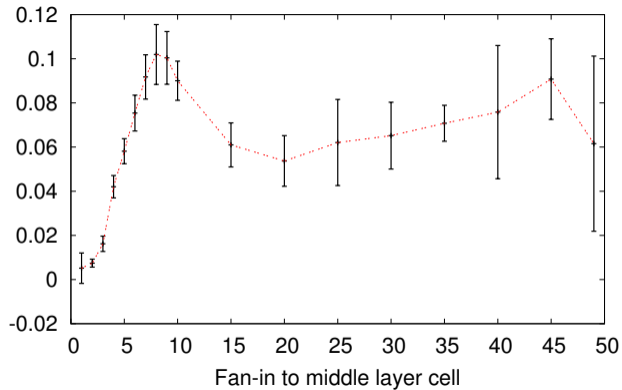

Supplement: S4 Fig — Performance of the model with respect to the varying fan-in from the input to the middle layer cells. Each point is a final performance averaged from 10 independent trials, each trial running for 2.104 epochs. Left: Simple task. From fan-in > 4, the model reached performance close to the optimum level. Right: Complex task. The learning performance was almost zero for fan-in < 4, gradually improving until it peaked around 8-9 and then decreasing slowly for even higher fan-in connectivity. (PDF) [file pcbi.1005705.s004.pdf]

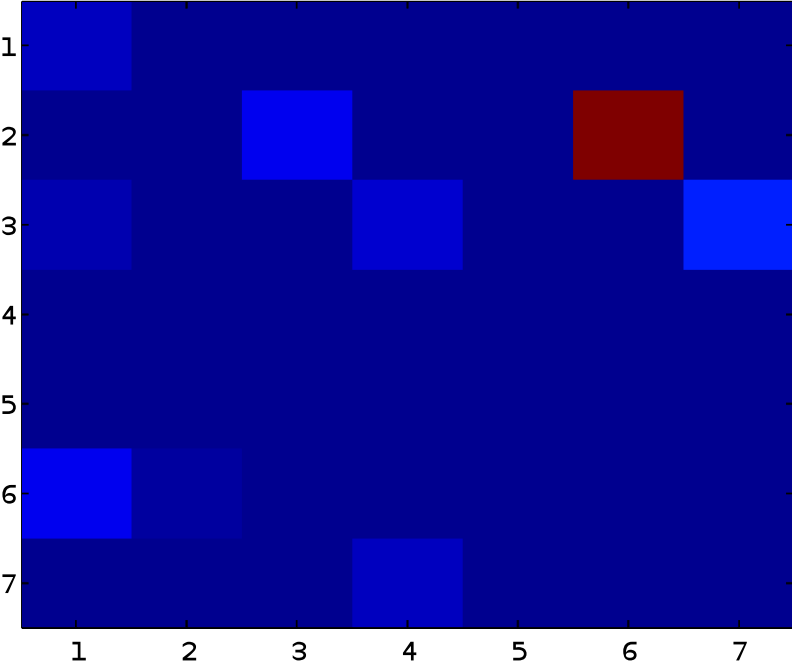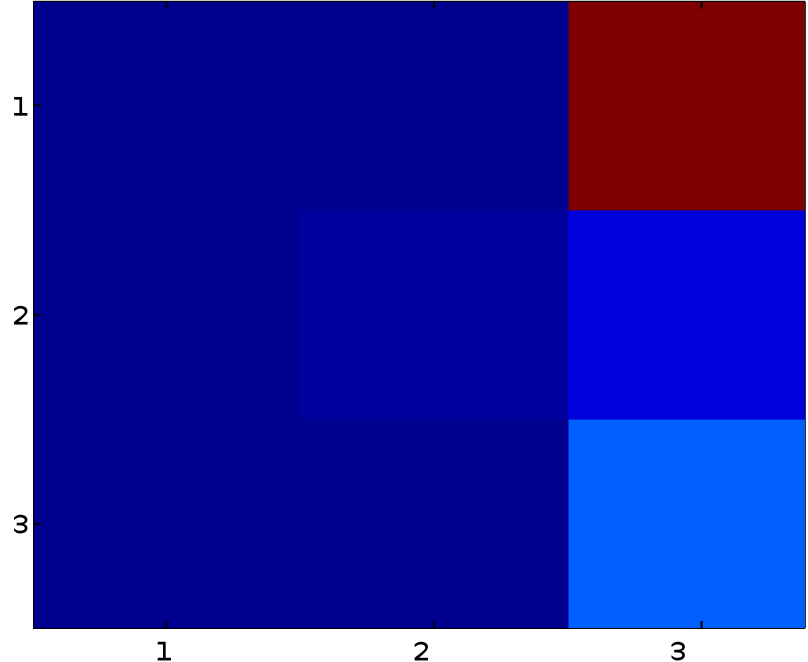

Supplement: S5 Fig — Strengths of the synaptic inputs (left) and outputs (right) of a typical middle layer neuron after successful training. The network has been trained to move toward any (single) food particle. Red represents the highest synaptic strength while blue represents the lowest strength. A characteristic middle layer cell that became responsive to a single food particle in the top right of the visual field (left) and excited the top right output cell (right) which moved the virtual agent toward the food particle. (PDF) [file pcbi.1005705.s005.pdf]
